# Tentacle Morphological Variation Coincides with Differential Expression of Toxins in Sea Anemones

**DOI:** 10.3390/toxins13070452

**Published:** 2021-06-29

**Authors:** Lauren M. Ashwood, Michela L. Mitchell, Bruno Madio, David A. Hurwood, Glenn F. King, Eivind A. B. Undheim, Raymond S. Norton, Peter J. Prentis

**Affiliations:** 1 School of Biology and Environmental Science, Faculty of Science, Queensland University of Technology, Brisbane, QLD 4000, Australia; d.hurwood@qut.edu.au (D.A.H.); p.prentis@qut.edu.au (P.J.P.); 2 Medicinal Chemistry, Monash Institute of Pharmaceutical Sciences, Monash University, 381 Royal Parade, Parkville, VIC 3052, Australia; michela.mitchell@qm.qld.gov.au (M.L.M.); ray.norton@monash.edu (R.S.N.); 3 Sciences Department, Museum Victoria, G.P.O. Box 666, Melbourne, VIC 3001, Australia; 4 Queensland Museum, P.O. Box 3000, South Brisbane, QLD 4101, Australia; 5 Bioinformatics Division, Walter & Eliza Hall Institute of Research, 1G Royal Parade, Parkville, VIC 3052, Australia; 6 Institute for Molecular Bioscience, The University of Queensland, St Lucia, QLD 4072, Australia; brunomadio@yahoo.com.br (B.M.); glenn.king@imb.uq.edu.au (G.F.K.); 7 Centre for Agriculture and the Bioeconomy, Queensland University of Technology, Brisbane, QLD 4000, Australia; 8 ARC Centre for Innovations in Peptide and Protein Science, The University of Queensland, St Lucia, QLD 4072, Australia; 9 Centre for Advanced Imaging, The University of Queensland, St Lucia, QLD 4072, Australia; eivind.a.b.undheim@ntnu.no; 10 Centre for Biodiversity Dynamics, Department of Biology, Norwegian University of Science and Technology, NO-7491 Trondheim, Norway; 11 Centre for Ecological and Evolutionary Synthesis, Department of Biosciences, University of Oslo, Blindern, NO-0316 Oslo, Norway; 12 ARC Centre for Fragment-Based Design, Monash University, Parkville, VIC 3052, Australia

**Keywords:** Actiniaria, venom, toxin expression, transcriptomics, ecology

## Abstract

Phylum Cnidaria is an ancient venomous group defined by the presence of cnidae, specialised organelles that serve as venom delivery systems. The distribution of cnidae across the body plan is linked to regionalisation of venom production, with tissue-specific venom composition observed in multiple actiniarian species. In this study, we assess whether morphological variants of tentacles are associated with distinct toxin expression profiles and investigate the functional significance of specialised tentacular structures. Using five sea anemone species, we analysed differential expression of toxin-like transcripts and found that expression levels differ significantly across tentacular structures when substantial morphological variation is present. Therefore, the differential expression of toxin genes is associated with morphological variation of tentacular structures in a tissue-specific manner. Furthermore, the unique toxin profile of spherical tentacular structures in families Aliciidae and Thalassianthidae indicate that vesicles and nematospheres may function to protect branched structures that host a large number of photosynthetic symbionts. Thus, hosting zooxanthellae may account for the tentacle-specific toxin expression profiles observed in the current study. Overall, specialised tentacular structures serve unique ecological roles and, in order to fulfil their functions, they possess distinct venom cocktails.

## 1. Introduction

Venom composition tends to be dynamic, varying across geographic locations and ontogenic stages, and between individuals in venomous taxa [1,2,3,4,5,6,7]. Phylum Cnidaria is considered the most ancient venomous lineage and is differentiated from most other venomous lineages by the fact the venom production does not occur within a central venom gland [8,9,10]. Instead, cnidae, the highly specialised organelles responsible for venom production and delivery, are found within ectodermal and endodermal tissue across the entire body plan [11,12,13], creating a unique link between toxin functional ecology and tissue functional morphology [9]. Indeed, differential expression of toxins across life stages and discrete anatomical regions has been reported across several taxa in the order Actiniaria (sea anemones) [13,14,15]. Different toxin profiles have been found in acrorhagi, tentacles, mesenterial filaments and body column [14,15], although these tissue-specific toxin expression profiles vary across species. For example, sea anemone sodium channel inhibitory toxins were observed to be upregulated in the column of *Anemonia sulcata* and *Heteractis crispa*, but in the mesenterial filaments of *Megalactis griffithsi* and the acrorhagi of *Actinia tenebrosa* [14,15]. However, multiple tentacle subtypes with distinct morphology and ecological roles may be present simultaneously within a species, suggesting that toxin expression profiles may be fine-tuned at an even greater level of detail.

While tentacles are used primarily for prey capture and defence against predators (feeding tentacles), contact with competitors can induce the development of a secondary tentacle type—catch tentacles—in actiniarians [16,17,18,19]. Analogous competitor-induced tentacles (sweeper tentacles) can be observed in scleractinian corals and octocorals [19,20,21,22]. Both catch and sweeper tentacles are specialised for agonistic encounters with competitors, causing necrosis in other polyps, and are morphologically distinct from feeding tentacles [20,22,23]. A recent investigation of gene expression in sweeper and feeding tentacles in the stony coral *Galaxea fasicularis* revealed that toxin genes are differentially expressed across the two tentacle subsets [24] indicating that venom composition may vary across tentacle types when they fulfil distinct ecological functions. We surmised that toxin expression might similarly differ in a tentacle-specific manner in sea anemones, given that they also possess functionally distinct tentacle subsets.

**Figure 1:**
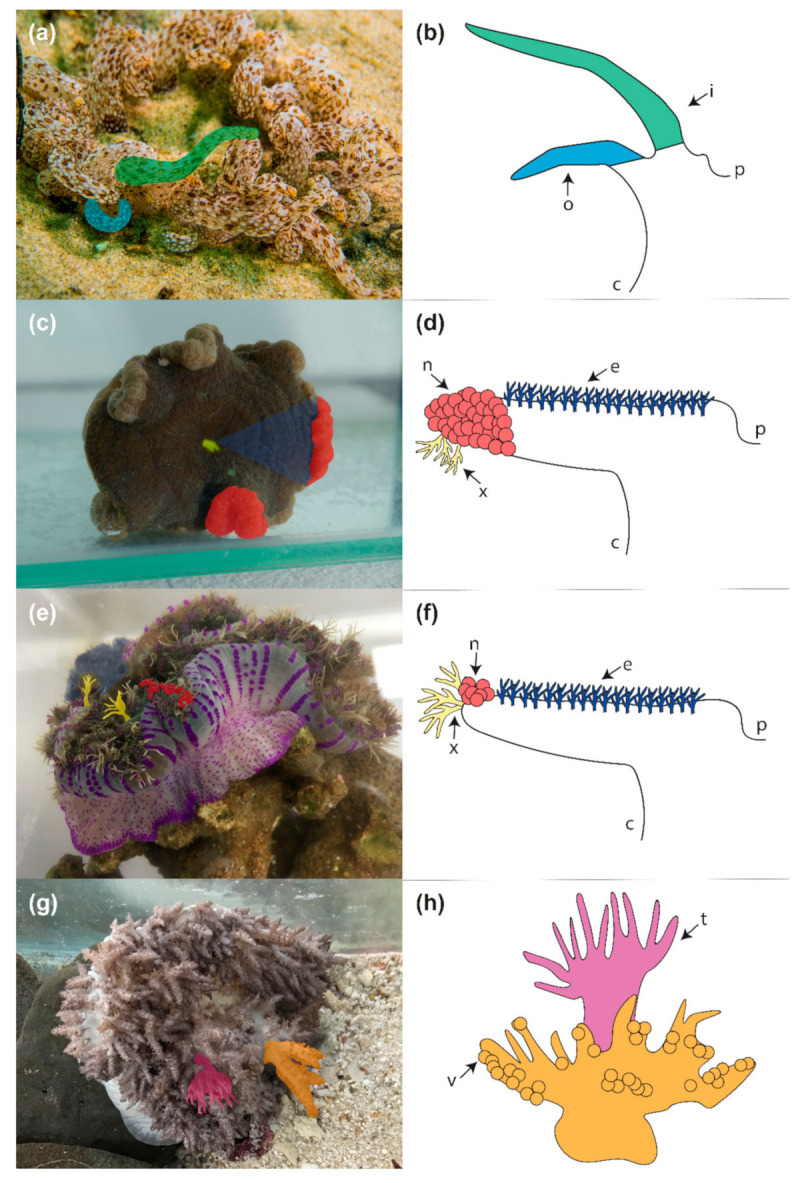
Tentacular structures isolated from sea anemone species: (**a**,**b**) inner tentacles (green) and outer tentacles (blue) from *D.* cf. *armata*; (**c**,**d**) endocoelic tentacles (dark blue) and nematospheres (red) from *C. adhaesivum*; (**e**,**f**) endocoelic tentacles (dark blue), exocoelic tentacles (yellow) and nematospheres (red) from *H. hemprichii;* (**g**,**h**) tentacles (pink) and pseudotentacles with attached vesicles (orange) from *P. semoni.* Abbreviations: c = body column; p = actinopharynx; I = inner tentacles; o = outer tentacles; n = nematospheres; x = exocoelic tentacles; e = endocoelic tentacles; v = vesicles on pseudotentacles; t = true tentacles. Photo credit: Gary Cranitch (**a**) and Lauren Ashwood (**c**,**e**,**g**).

Actiniarian tentacle morphology is highly variable, and several species possess additional specialised tentacular structures [19,25,26,27]. While tentacles in many species are characterised by simple external morphology, they can vary in structure (e.g., branched) or possess nematocyst-dense structures [28]. The relative size of tentacles can also vary, as observed with the long inner and short outer tentacles of genera *Dofleinia* and *Macrodactyla* (Figure 1a,b). Additionally, *Dofleinia armata* has a discrete battery of cells (papillae) covering their tentacles which are laden with nematocysts [29]. Tentacles co-locate with mesenterial spaces, which are termed endocoels or exocoels depending on whether they arise inside mesenterial pairs or between mesenterial pairs, respectively [30]. In many actiniarian taxa, a single tentacle is associated with each endocoel/exocoel, although in *Cryptodendrum adhaesivum* and *Heterodactyla hemprichii* multiple tentacles are associated with each endocoel (Figure 1c–f), with a clear morphological distinction between endocoelic and exocoelic tentacles [26,31,32]. Exocoelic tentacles of *C. adhaesivum* and *H. hemprichii* are orally-aborally flattened, branched structures present at the margin of the oral disc [30,31]. In contrast, dendritic endocoelic tentacles of these species are arranged in rows that radiate out from the mouth and are considerably shorter than exocoelic tentacles [31,32].

In addition to endocoelic and exocoelic tentacles, species from the family Thalassianthidae possess a third tentacle type: nematospheres (Figure 1c–f). Characterized by a spherical morphology, these modified tentacles are taxonomically restricted, although similar globular structures can be observed in other species [26,32,33]. While associated with an endocoel, nematospheres are co-located with exocoelic tentacles at the oral disc margin but likely serve different ecological functions [26].

In *H. hemprichii*, nematospheres form distinctive grape-like clusters, while in *C. adhaesivum* the nematospheres form a continuous band adjacent to endocoelic tentacles, although the boundary between the two tentacle types is clearly defined [26,34]. The function of nematospheres has not yet been determined but they are presumed to serve a defensive role [26]. Vesicles in *Phyllodiscus semoni* are comparably nematocyst-dense spherical structures attached to the pseudotentacles (Figure 1g,h) [26]. Pseudotentacles are restricted to the family Aliciidae and differ from true tentacles in that they are outgrowths of the lower column wall [26,35]. Tentacles and pseudotentacles alternate, extending and retracting in a cyclic manner, with pseudotentacles extending and obscuring the true tentacles during the day [35,36]. The alternating retraction of pseudotentacles and tentacles implies that these structures have specific functions, which may require different venom compositions, resulting in differential expression of toxins across these morphological features.

Given that differences in the functional profiles of structures correlate to the differential expression of toxins in a tissue-specific manner [14,15,24], distinct tentacle-specific toxin expression patterns may be present across tentacle subtypes that serve different ecological functions. Furthermore, there is likely to be a functional basis for morphological variants of tentacles observed in Aliciidae and Thalassianthidae. In this study, we assessed whether morphological variants of tentacles are associated with distinct toxin expression profiles and investigated the functional significance of specialised tentacular structures. Our results provide insight into the underlying morphological basis for the different toxin profiles associated with functionally distinct tissues.

## 2. Results

### 2.1. Assembly Statistics

We sequenced and assembled the transcriptomes of five sea anemone species collected from Australian waters: *C. adhaesivum*, *D.* cf. *armata*, *H. hemprichii*, *M. doreensis* and *P. semoni*. More than 142 million paired-end reads were generated for *M. doreensis*, *C. adhaesivum*, *D.* cf. *armata* and *P. semoni*, while 240 million single-end reads were produced for *H. hemprichii*. The library for endocoelic tentacles of *C. adhaesivum* appeared to have degraded during transport and was not able to be sequenced. Transcriptomes for all taxa had Benchmarking Universal Single-Copy Orthologs (BUSCO) completeness scores between 88.9 and 97.2%. The assembly N50 scores ranged from 609 to 2823 base pairs (bp). Although the N50 for *C. adhaesivum* (609; Table 1) was significantly lower than the other species, the N50 calculated from only the most highly expressed transcripts (1346; Table 1) was comparable to that of *M. doreensis* (1493; Table 1).

### 2.2. Functional Differences of Structures

**Figure 2:**
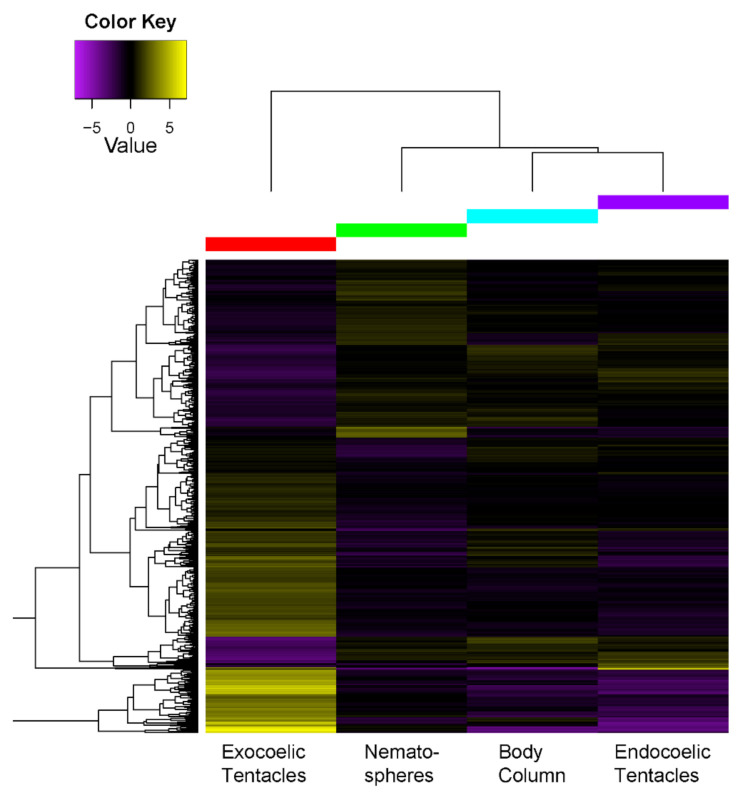
Heatmap of differentially expressed genes (centered reads per kilobase of transcript per million mapped [RPKM] values) for body column and tentacular structures in *H. hemprichii*.

Differential gene expression and Gene Ontology (GO) enrichment analysis supported functionally discrete roles for the structures analysed. Patterns of transcript expression differed significantly across the structures examined in all taxa. When comparing gene expression between inner and outer tentacles of *D.* cf. *armata* and *M. doreensis*, 156 and 271 transcripts were found to be differentially expressed, respectively. Higher numbers of differentially expressed transcripts were observed among structures in *C. adhaesivum*, *H. hemprichii* and *P. semoni* (Table 1). In *H. hemprichii*, transcript expression in the body column showed the most divergent expression pattern, with 978, 1050 and 1996 genes differentially expressed between the body column and endocoelic tentacles, exocoelic tentacles and nematospheres, respectively (Figure 2). Comparing expression profiles among tentacle types, we found that exocoelic tentacles and nematospheres, which are both located at the marginal edge of the oral disc, had the highest degree of similarity with 327 genes differentially expressed. Conversely, 824 genes were differentially expressed between the endocoelic tentacles and nematospheres. Comparisons between two structures in the remaining species revealed that fewer transcripts were differentially expressed between tentacular structures in *P. semoni* (1411) than between the body column and tentacles in *C. adhaesivum* (3672). However, in both species, comparable numbers of transcripts were upregulated in each of the structures examined (Figure S1).

GO enrichment analysis of differentially expressed genes also supported greater differences among tentacular structures of *H. hemprichii* and *P. semoni* than between inner and outer tentacles of *D.* cf. *armata* and *M. doreensis.* Extracellular region (GO:0005576) was found to be enriched in outer tentacles of *D.* cf. *armata* and *M. doreensis*. While no GO terms were enriched for inner tentacles of *D.* cf. *armata,* photosynthesis (GO:0015979) and related terms were enriched in inner tentacles of *M. doreensis.* Similarly, chlorophyll binding (GO:0016168) was enriched in nematospheres of *C. adhaesivum* and vesicles of *P. semoni*. The enrichment of photosynthesis-related terms likely reflects the presence of algal symbionts in *C. adhaesivum*, *M. doreensis* and *P. semoni*. *Heterodactyla hemprichii* also hosts zooxanthellae species, although the specimen included in this study had undergone bleaching prior to tissue dissection and therefore no photosynthetic-related GO terms were found to be enriched in this individual. Venom-related GO terms, including nematocyst (GO:0042151) and toxin activity (GO:0090729), were significantly enriched in multiple structures across *C. adhaesivum*, *H. hemprichii* and *P. semoni*.

### 2.3. Venom Repertoire and Toxin Expression

#### 2.3.1. Comparison of Species Venom Arsenal

Transcripts with homology to known toxins (toxin-like) were identified and extracted from the transcriptome assembly of each taxon. The number of toxin-like transcripts compiled for each species ranged from 60 in *H. hemprichii* to 119 in *C. adhaesivum* and *P. semoni* (Table 2), with approximately 0.05% of transcripts categorised as toxin-like.

**Figure 3:**
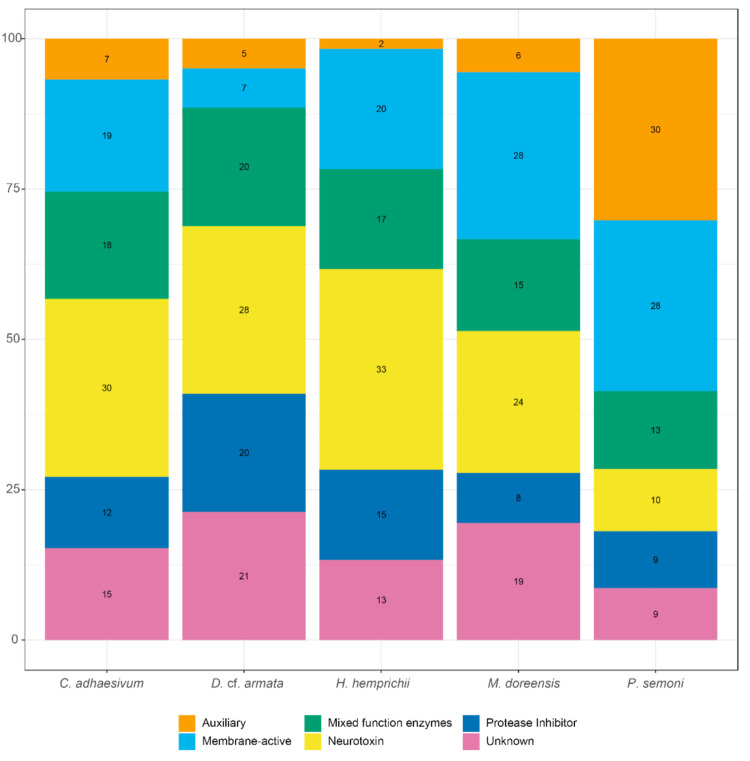
Composition of toxin arsenal for *C. adhaesivum*, *D.* cf. *armata*, *H. hemprichii*, *M. doreensis* and *P. semoni*. The functional classes of toxins are presented as percentage of identified toxin-like transcripts.

In order to summarise the distinct venom profile of each actiniarian species, toxin-like transcripts were categorised into toxin families and then assigned one of six biological functions: auxiliary, membrane-active, mixed function enzymes, neurotoxin, protease inhibitor and unknown (Table 2 and Figure 3). Toxin families with neurotoxic functions were associated with the largest number of toxin-like transcripts in *C. adhaesivum*, *D.* cf. *armata* and *H. hemprichii*. In particular, sea anemone type 3 (BDS) potassium channel toxin-like transcripts were numerous in all three species. Conversely, membrane-active toxins were the most abundant category of toxins identified in the transcriptome of *M. doreensis*. The Membrane Attack Complex/Perforin (MACPF) toxin family is expanded in this species, with MACPF toxin-like transcripts also highly abundant in the transcriptome of *P. semoni*. Auxiliary toxins comprised a much higher proportion of the venom arsenal in *P. semoni* compared to the other four species; this can be explained by the large number of toxin-like transcripts with homology to peptidase M12A.

#### 2.3.2. Tissue-Specific Expression of Toxin-Like Transcripts

At least one toxin-like transcript was differentially expressed across tentacle types for all species. Mirroring the pattern observed for all transcripts, the number of toxin-like transcripts differentially expressed across tentacle types was greatly reduced in *D.* cf. *armata* and *M. doreensis* compared to all other species. Only a single toxin-like transcript was found to be significantly differentially expressed (4-fold, false discovery rate [FDR] < 0.05) between inner and outer tentacles. The toxin-like transcript that was upregulated in the inner tentacles of *D.* cf. *armata* has high sequence similarity to nematocyst expressed protein 6 (K7Z9Q9), a member of the peptidase M12A toxin family (Figure S2a). Conversely, in *M. doreensis*, the toxin-like transcript had a significant BLASTp hit to δ-actitoxin-Amc1a (P69929), a boundless beta-hairpin type sea anemone neurotoxin (Figure S2b) [37]. Therefore, only minimal differential expression of toxins is observed in actiniarian species examined here whose tentacles are characterised by a difference in size rather than morphology.

**Figure 4:**
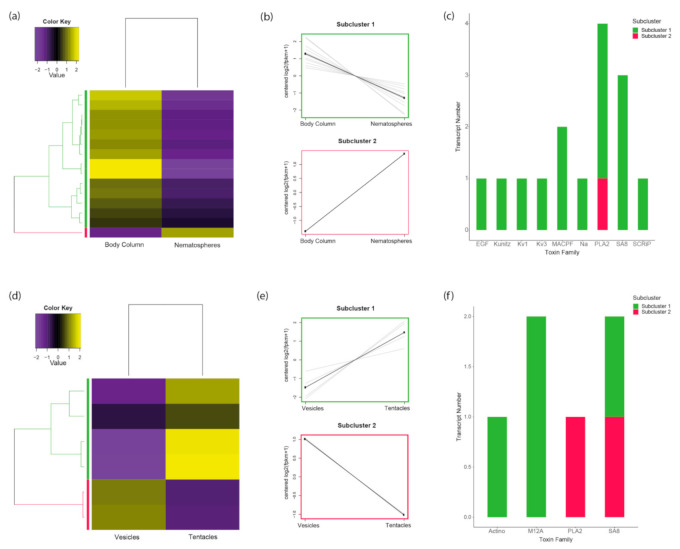
Toxin expression profiles of tentacular structures in *C. adhaesivum* and *P. semoni*: (**a**) Heatmap of differentially expressed toxin genes (centered fragments per kilobase of transcript per million mapped [FPKM] values) for *C. adhaesivum*; (**b**) Plot of the expression profile for each subcluster of differentially expressed toxin-like transcripts for *C. adhaesivum*; (**c**) Bar plot of toxin family distribution across subclusters for *C. adhaesivum*; (**d**) Heatmap of differentially expressed toxin genes (centered FPKM values) for *P. semoni*; (**e**) Plot of the expression profile for each subcluster of differentially expressed toxin-like transcripts for *P. semoni*; (**f**) Bar plot of toxin family distribution across subclusters for *P. semoni*. Abbreviations: Actino = Actinoporin; EGF = endothelial growth factor domain peptide; Kunitz = Venom Kunitz-type; Kv1 = Sea anemone type 1 potassium channel toxin; Kv3 = Sea anemone type 3 (BDS) potassium channel toxin; M12A= Peptidase M12A; MACPF= Membrane-attack complex/perforin; Na = Sea anemone sodium channel inhibitory toxin; PLA2 = Phospholipase A_2_; SA8 = Sea anemone 8 toxin; SCRiP= Cnidaria small cysteine-rich protein.

In *C. adhaesivum*, 15 toxin-like transcripts were differentially expressed between the body column and nematospheres (Figure 4a–c). Cutting the dendrogram at 50% of its height led to partitioning of the toxin-like transcripts into two subclusters: 14 transcripts were upregulated in the body column (subcluster 1) and one transcript was upregulated in nematospheres (subcluster 2). Eight toxin families were upregulated only in the body column, including multiple MACPF-like toxins. 57% of these toxin-like transcripts were neurotoxins or toxins with unknown function. Several genes from the phospholipase A_2_ family were upregulated in both the body column and nematospheres of this individual. Thus, a more diverse profile of toxins is upregulated in the body column and the toxin significantly upregulated in the nematospheres is enzymatic in nature.

In the single *P. semoni* specimen examined, six toxin-like transcripts were differentially expressed between the true tentacles and vesicles (Figure 4d–f). When cut at 50% of its height, the hierarchically clustered gene tree splits into two subclusters: four transcripts were upregulated in tentacles (subcluster 1) and two transcripts were upregulated in vesicles (subcluster 2). Within the tentacles, toxin families associated with auxiliary, membrane-active and unknown functions were found to be upregulated. Toxin-like transcripts consistently upregulated only in vesicles have homology to phospholipase A_2_. Only the sea anemone 8 toxin family is upregulated in both the vesicles and tentacles. Overall, these findings reinforce that toxin expression is more likely to diverge when substantial morphological variation is present among tentacular structures.

**Figure 5:**
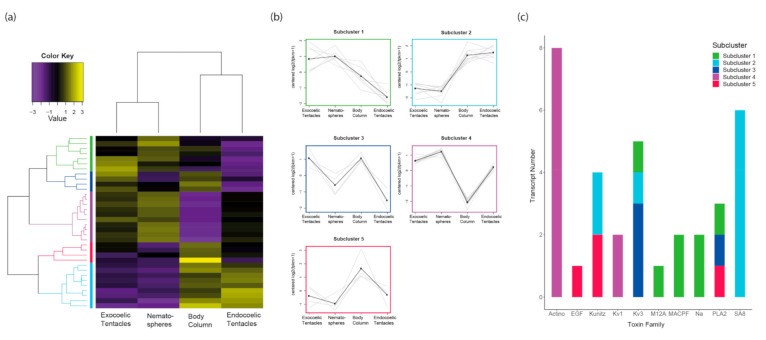
Toxin expression profiles of tentacle structures in *H. hemprichii*: (**a**) Heatmap of differentially expressed toxin genes (centered RPKM values) for *H. hemprichii*; (**b**) Plot of the expression profile for each subcluster of differentially expressed toxin-like transcripts for *H. hemprichii*; (**c**) Bar plot of toxin family distribution across subclusters for *H. hemprichii*. Abbreviations: Actino = Actinoporin; EGF = endothelial growth factor domain peptide; Kunitz = Venom Kunitz-type; Kv1 = Sea anemone type 1 potassium channel toxin; Kv3 = Sea anemone type 3 (BDS) potassium channel toxin; M12A= Peptidase M12A; MACPF= Membrane-attack complex/perforin; Na = Sea anemone sodium channel inhibitory toxin; PLA2 = Phospholipase A_2_; SA8 = Sea anemone 8 toxin; SCRiP= Cnidaria small cysteine-rich protein.

In *H. hemprichii*, 34 toxin-like transcripts were differentially expressed across the four morphological features: body column, endocoelic tentacles, exocoelic tentacles and nematospheres (Figure 5). A high degree of similarity is observed between exocoelic tentacles and nematospheres, even when examining only transcripts with significant homology to known toxins. Unlike the heatmap for all transcripts, the body column clusters with the endocoelic tentacles when considering only expression of toxin-like transcripts. This suggests that while the body column and endocoelic tentacles are distinct anatomical regions, the venom from both structures may serve similar ecological functions.

Furthermore, a relatively small number of toxin-like transcripts was differentially expressed between the body column and endocoelic tentacles of this individual (10). More toxin-like transcripts were differentially expressed between endocoelic tentacles and nematospheres (15) than between endocoelic tentacles and exocoelic tentacles (13). Overall, the highest number of differentially expressed toxin-like transcripts was observed when comparing the body column and nematospheres (24), and the lowest when comparing exocoelic tentacles and nematospheres (1).

Cutting the dendrogram at 50% of its height led to partitioning of the toxin-like transcripts into five subclusters with the following expression patterns: upregulated in exocoelic tentacles and nematospheres (subcluster 1, seven transcripts); upregulated in the body column and endocoelic tentacles (subcluster 2, nine transcripts); upregulated in exocoelic tentacles and body (subcluster 3, 4 transcripts); upregulated in all tentacle types (subcluster 4, 10 transcripts); and upregulated in the body column (subcluster 5, four transcripts).

When examining the subclusters in conjunction with the biological function of toxins, a pattern emerged in that a single functional category was generally not present in more than two subclusters. The exceptions to this were neurotoxins and mixed function enzymes, which were present in four and three subclusters, respectively. Interestingly, auxiliary function toxin-like transcripts were only found in cluster 5, while the two membrane-active toxin families were consistently upregulated across exocoelic tentacles and nematospheres. Further interrogating the differential expression data, we established that 5, 3, 2, 2, and 3 toxin protein families were found in subclusters 1, 2, 3, 4 and 5, respectively. Additionally, many toxin families were found to be isolated within a single subcluster. Sea anemone type 3 (BDS) potassium channel and phospholipase A_2_ toxins were found in three subclusters, the most of any toxin family. Thus, most toxin families are associated with distinct expression patterns across the envenomating structures examined in *H*. *hemprichii.*

## 3. Discussion

### 3.1. Tissue-Specific Toxin Expression Profiles of Tentacles Show Greater Divergence When Morphological Variation Is Present

Differential expression of toxins between discrete anatomical regions has been established in the actiniarian families Actiniidae, Actinodendridae and Stichodactylidae [14,15]. Likewise, we report the differential expression of toxins between the body column and tentacles in a species from Thalassianthidae. Further building on this, we also report that toxin expression can vary significantly across morphologically disparate tentacle types. Comparing expression profiles of morphologically similar inner and outer tentacles, only a single toxin-like transcript was found upregulated for each species from Actiniidae. The inner and outer tentacles of *D.* cf. *armata* and *M. doreensis* are simple unbranched tentacles, differentiated only by size, indicating that morphologically similar tentacles have comparable toxin profiles. As different cnidae type are associated with different venom profiles in cnidarians [13,38], the minimal differences in toxin expression across inner and outer tentacles may suggest that the cnidome does not vary significantly across these tentacles within *D.* cf. *armata* and *M. doreensis*. However, more pronounced differences in gene expression are observed when substantial morphological variation exists within tentacles.

Members of Thalassianthidae possess three morphologically distinct types of tentacles: endocoelic tentacles, exocoelic tentacles and nematospheres [26]. Endocoelic and exocoelic tentacles are branched outgrowths located in rows radiating out from the mouth and along the margin of the oral disc, respectively [26,31,32]. Nematospheres are a specialised form of endocoelic tentacles, characterised by a spherical morphology and high nematocyst density, observed exclusively in Thalassianthidae [26]. Comparison of toxin expression across morphologically distinct tentacle types in one *H. hemprichii* individual indicates that endocoelic tentacles, exocoelic tentacles and nematospheres are each associated with a unique toxin profile. Differences in toxin expression between endocoelic and the other tentacle types is more pronounced than between exocoelic tentacles and nematospheres. Tissue-specific toxin expression with similar profiles have been observed between functionally similar structures, such as acrorhagi and tentacles in *A. tenebrosa* [14,15]. Given the high degree of similarity in their toxin expression, it is likely that venom produced by exocoelic tentacles and nematospheres fulfils similar functional demands in *H. hemprichii*.

### 3.2. Ecological Significance of Tentacular Structures, and Its Relationship with Toxin Expression Profiles

The location of nematospheres and exocoelic tentacles at the oral disc margin may provide insight into some of the functional demands of these two tentacle types. Actiniarians are soft-bodied creatures that rely on venom for multiple ecological functions, including defence against predators [9,25]. Nevertheless, behavioural responses, such as the retraction of tentacles, can also offer protection from predation [28]. In many actiniarian species, prolonged contact with the nudibranch species *Berghia stephania* resulted in fully retracted tentacles being concealed by the sphincter [28,39]. However, concealment of tentacles is not possible in *Anemonia sulcata* due to anatomical differences [39] and is unlikely to occur in a species with an expanded oral disc, such as *C. adhaesivum* and *H. hemprichii.* However, it has been observed that the oral disc of *C. adhaesivum* expands and contracts in response to light conditions. When exposed to low light conditions, the oral disc folds in on itself, with the region bearing nematospheres curling towards the mouth and concealing the majority of endocoelic tentacles [40]. Therefore, it stands to reason that venom from nematospheres and exocoelic tentacles must contain toxins which would deter predators, possibly through pain induction [41].

Similarly, vesicles and pseudotentacles in species from Aliciidae are predicted to have a major role in predator deterrence. During the day, tentacles are retracted in *P. semoni* and offer no protection against predators [35,36]. Conversely, vesicles and pseudotentacles extend in response to light exposure, obscuring the retracted tentacles [36], and therefore these are the structures that predators are most likely to come into contact with. While technically not a tentacle structure, as they arise from the body column [26], these “tentacle-like” structures appear to be primarily responsible for defence against predators, a function typically assigned to true tentacles. However, *P. semoni* participates in predatory feeding behaviour at night when the tentacles are extended [35,36], so venom from the tentacle-like structures is unlikely to play a major role in prey capture. The significant differences in the toxin expression profiles of *P. semoni* tentacle and tentacle-like structures can be explained by the discrete key roles of these structures in predatory and defensive behaviour, respectively. Thus, nematospheres and vesicles share a common functional role in defence, but these structures are taxonomically restricted within two different actiniarian superfamilies (Actinioidea and Metridioidea, respectively) [26,42] and likely differ in their venom content and anatomy.

Phylogenetic analysis conducted by Crowther [26] demonstrated that the spherical defensive structures (nematospheres and vesicles) present in Thalassianthidae and Aliciidae are superficially similar but not homologous structures. The nematocyst content of the nematospheres and vesicles has been found to differ [26] and we report that very few toxin families are upregulated across both morphological features. Even within the same structure across different species, toxin expression profiles differ substantially in actiniarians [15]. Within nematospheres of *C. adhaesivum* we find an enzymatic toxin family upregulated. Conversely, in *H. hemprichii*, membrane-active toxins and neurotoxins are the predominant functional classes of toxins significantly upregulated in the nematospheres compared to other structures. Variation in the cnidome of tentacular structures between species may account for these differences, with different cnidae types associated with different venom profiles in cnidarians [13,38]. Basitrichs are common across the tentacles of *C. adhaesivum* and *H. hemprichii* but vary in size, with the largest basitrichs observed in the nematospheres [26,43,44]. While microbasic p-mastigophores occur in endocoelic tentacles of both species, they are abundant in *C. adhaesivum* but rare in *H. hemprichii* [26,44,45]. Additionally, greater diversity of cnidae is present in the exocoelic tentacles of *C. adhaesivum* relative to *H. hemprichii* [26,44]. These differences in the type, size, and abundance of cnidae probably all contribute to differences in the toxin profile of a tentacular structure across species, but the correlation between the cnidome of tentacular structures and their toxin profiles needs to be directly examined in future research. Therefore, while both nematospheres and vesicles function as defensive structures, the toxin cocktails they utilise differ between the two structures and even within a single structure across species.

### 3.3. Unique Toxin Arsenals Are Required by Structures That Defend Endosymbiont-Hosting Structures

Regionalisation of venom production has been observed in other venomous taxa, but in all instances there is an underlying functional basis for producing multiple venoms with distinct compositions. For example, the assassin bug *Pristhesancus plagipennis* and the cone snail *Conus geographus* produce distinct venoms specialised for predation and defence [46,47]. Differences in toxin expression across sweeper and feeding tentacles in corals can likewise be explained by the discrete roles of these two structures in intraspecific combat and prey capture, respectively [24]. While ecological interactions have minimal impact on the toxin gene complement in cnidarian species, they are known to function as keys drivers of toxin gene expression [14,25,48,49]. Consequently, understanding which ecological factors have driven *C. adhaesivum*, *H. hemprichii* and *P. semoni* to possess morphologically diverse tentacular structures will provide insights into the functional basis of the tentacle-specific toxin expression profiles observed in the current study.

Crowther demonstrated that convergent morphological adaptations can be observed in distantly related sea anemone families due to similar ecological demands [26]. Actiniarians are predominantly predatory, although many also rely upon zooxanthellae, intracellular algae symbionts, for nutrients [28]. Branched outgrowths, such as endocoelic tentacles and pseudotentacles, increase surface area, and thereby enable sea anemones to house more zooxanthellae and increase their photosynthetic capacity by increasing access to light [36]. Unfortunately, the *H. hemprichii* specimen used to generate the transcriptomic dataset had bleached, but the enrichment of photosynthetic-related gene GO terms in the nematospheres of *C. adhaevisum* supports the presence of zooxanthellae in this structure. The endoderm of tentacles in *C. adhaevisum* and *H. hemprichii* has previously been noted to contain a high proportion of zooxanthellae compared to other regions [26]. Zooxanthellae are similarly reported to be dense in the pseudotentacles of Aliciidae species [26] and photosynthetic-related GO terms were found to be enriched in the vesicles of *P. semoni*. Therefore, morphological diversity of tentacles supports hosting of endosymbionts in the families Thalassianthidae and Aliciidae.

Defensive spheres have convergently evolved at least three times in Actiniaria [26], resulting in nematospheres in Thalassianthidae, vesicles in Aliciidae and Boloceroididae, and acrospheres in Actinodendridae and Haloclavidae [26,50,51]. Species from the Aliciidae and Actinodendridae families can pose a significant health risk to humans, with envenomation by *P. semoni* and *Actinodendron plumosum* resulting in severe skin ulceration [52,53,54,55,56]. The painful sting of species from Actinodendridae is why they are collectively known as hell’s fire anemones [57,58], while an instance of death following envenomation by *P*. *semoni* has been reported [59]. The potent venom associated with nematospheres and vesicles, which is likely utilised to deter vertebrate predators, in combination with the exposed position of these structures when the oral disc contracts and tentacles retract, ensures these Aliciidae and Thalassianthidae are well armed against the threat of predation. However, the morphology and unique toxin profiles of these structures also serves to protect tissue where endosymbionts are housed in *C. adhaesivum, H. hemprichii* and *P. semoni,* and thereby protects a dependable energy source. Thus, hosting zooxanthellae appears to underpin both the morphological variation in tentacular structures and the distinct toxin profiles associated with each tentacular subtype in the subtidal species examined.

Acrospheres are swellings of the tentacles, characterised by a unique cnidome, which are present in orders Actiniaria and Corallimorpharia [26,50,51,60,61]. Within actiniarians, acrospheres represent a third spherical tentacle structure specialised for defence and can be observed in *Actinodendron* species and the haloclavid genera *Anemonactis* and *Haloclava* [26,51]. Additionally, the genus *Telmatactis* is defined by club-like swellings at the distal portion of tentacles [62] and, while not classified as acrospheres, these club-tips may serve a defensive function. Given the patterns of toxin expression of nematospheres and vesicles observed in the current study, acrospheres and indeed other morphologic variants of tentacles may also be associated with unique toxin profiles driven by underlying functional demands of the structures and therefore warrant further investigation. Obtaining multiple specimens of *C. adhaesivum*, *H. hemprichii* and *P. semoni* proved challenging and thus we were unable to verify findings across individuals from the same species. Future research should employ biological replicates to determine whether the tissue-specific toxin expression profiles associated with tentacular structures is subject to intraspecific variability. Furthermore, investigating the phylogenetic relationship of toxin families differentially expressed across tentacular structures may also provide greater insights into venom diversity within order Actiniaria.

## 4. Materials and Methods

### 4.1. Sample Preparation and Sequencing

#### 4.1.1. *Heterodactyla hemprichii*, *Cryptodendrum adhaesivum* and *Phyllodiscus semoni*

*Heterodactyla hemprichii*, *Cryptodendrum adhaesivum* and *Phyllodiscus semoni* were collected by Cairns Marine Pty Ltd. from the Great Barrier Reef, QLD, Australia and housed in holding tanks at the Queensland University of Technology under standard aquarium conditions [63]. Following a 72-h starvation period, anatomically distinct structures were dissected from a single individual for each species, flash frozen and stored at 80 °C for transcriptome sequencing. For *H. hemprichii*, tissue from the body column, endocoelic tentacles, exocoelic tentacles and nematospheres was isolated (Figure 1e). Tissue from the body column, endocoelic tentacles and nematospheres was also isolated from *C. adhaesivum* (Figure 1c). For *P. semoni,* tissue was isolated from the tentacles, pseudotentacles and vesicles (Figure 1g). However, due to the small size of the individual, the pseudotentacles and vesicles were dissected together and not separated for RNA extraction.

Total RNA was extracted from homogenised tissue using the TRIzol/chloroform protocol [64] followed by purification with an ISOLATE II RNA mini kit (Bioline, Sydney, Australia). RNA integrity and quantity were determined using a Bioanalyser 2100 (Agilent, Santa Clara, CA, USA) and NanoDrop 2000 Spectrophotometer (Thermo Fisher Scientific, Carlsbad, CA, USA). Sequencing libraries for *H. hemprichii* were prepared using a TruSeq RNA Library Prep Kit v2 (Illumina, San Diego, CA, USA) and sequenced using 75 bp single-end chemistry on an Illumina NextSeq 500. Sequencing libraries for *C. adhaesivum* and *P. semoni* were prepared using TruSeq Stranded mRNA Library Prep (Illumina, San Diego, CA, USA) and sequenced using 150 bp paired-end chemistry on an Illumina HiSeq X10.

#### 4.1.2. *Dofleinia* cf. *armata*

*Dofleinia* cf. *armata* (identified by sea anemone taxonomist M.L.M.) was collected from Southport, QLD, Australia under Queensland Museum permit 160782. After a 72-h starvation period, inner and outer tentacles (Figure 1a) from two individual specimens of *Dofleinia* cf. *armata* were collected and preserved in RNA*later* (Thermo Fisher Scientific, Carlsbad, CA, USA). Preserved tissue was lysed using a TissueLyser II (Qiagen, Hilden, Germany) and total RNA extracted using a QIAGEN RNeasy Mini Kit. Extracted RNA with an integrity number (RIN) above 8.5 was used for library preparation. Individual cDNA libraries were prepared using PolyA-enriched RNA-seq stranded sample prep (Illumina, San Diego, CA, USA). Libraries were sequenced using 100 bp, paired-end sequencing on an Illumina HiSeq 2500. Preserved animals (QM G335858 & QM G335860) are lodged in the Queensland Museum. Full details of *D.* cf. *armata* extraction and sequencing methods have been published previously [65].

#### 4.1.3. *Macrodactyla doreensis*

Two specimens of *Macrodactyla doreensis* were collected from North Stradbroke Island, QLD, Australia under the permit QS2014/MAN259 and kept in aquaria at The University of Queensland. Tissue from inner and outer tentacles from two individual specimens was collected with tweezers and flash frozen before total RNA was extracted using TRIzol (Thermo Fisher Scientific, Carlsbad, CA, USA) and enriched for mRNA using a DynaBeads Direct mRNA kit (Thermo Fisher Scientific, Carlsbad, CA, USA) as described [66]. cDNA libraries for each sample were then prepared using a TruSeq library kit (Illumina, San Diego, CA, USA) and sequenced on an Illumina NextSeq 500, using 150 bp paired-end chemistry.

### 4.2. Transcriptome Assembly

Raw reads were quality checked using FastQC (http://www.bioinformatics.babraham.ac.uk/projects/fastqc/, accessed on 23 March 2018) and tissue-specific reads were pooled prior to transcriptome assembly for each species. Sequence reads were filtered (Q > 30, N < 1%), with non-biological sequences and low-quality reads removed using Trimmomatic [67]. The Trinity short read de novo assembler v2.5.1 was used to assemble the remaining high-quality reads into contigs using default parameters for the specific sequencing libraries, with strand-specific RNA-seq read orientation provided for stranded assemblies [68].

Post-assembly, redundant contigs (≥98% similarity) were removed using CD-Hit EST v4.6.4 [69]. BUSCO v3.0.1 was used to assess the completeness of each assembly [70]. Additionally, Trinity scripts were used to compute contig N50 and Ex90N50 statistics for all assemblies.

*M. doreensis* and species from the families Thalassianthidae (*C. adhaesivum* and *H. hemprichii)* and Aliciidae (*P. semoni*) are known to contain symbiotic dinoflagellate algae [26,35,71]. Therefore, the PsyTrans Python script (https://github.com/sylvainforet/psytrans, accessed on 5 April 2017) was employed to remove potential symbiont contamination from the assembled transcriptomes of *M. doreensis* and *H. hemprichii*. Host reference sequences were derived from the *Nematostella vectensis* genome proteins models [72]. The combined genome protein models of *Symbiodinium microadriaticum*, *S*. *minutum* and *S*. *kawagutii* [73,74,75] served as symbiont reference sequences. Default script settings were used to analyse the sea anemone transcriptomes, except for the following script modifications; maxBestEvalue = 1e-30, numberOfSeq = minimum blast hit count, Line 55: Training Proportion = 1.0, Line 566: if nSeqs < options.args.numberOfSeq:, Line 572: for i in xrange(trainingMaxIdx, options.args.numberOfSeq):.

### 4.3. Tissue-Specific Expression Profiles

#### 4.3.1. Functional Annotation of Transcripts

Functional annotation was conducted using the Trinotate protocol. Open reading frames (ORFs) encoding more than 65 amino acid residues were extracted from assemblies using TransDecoder 3.0.1 [76]. To determine homology to known proteins, translated ORFs were used as BLASTp queries against the UniProt database (accessed on 2 June 2020). Additionally, contigs were used as BLASTx queries against the UniProt database. Significant BLAST hits were defined by a threshold e-value of 1 × E^−5^. Protein family (Pfam) domains were identified using HMMER 3.1b2 software [77]. Using Trinotate [76], GO terms associated with both BLAST and Pfam domain hits were generated.

#### 4.3.2. Differential Expression Analysis and Gene Ontology Enrichment Analysis

Bowtie2 was used to align raw reads to assembled contigs [78], followed by transcript quantification with RSEM 1.2.30, using a strand-specific flag for *C. adhaesivum* and *P. semoni* [79]. Differential gene expression was calculated using RSEM outputs and the edgeR Bioconductor package v3.3.1, with the dispersion value set to 0.1 when no biological replicates were present [80]. Using the Trinity suite, differentially expressed transcripts (4-fold, FDR ≤ 0.05) were extracted and clustered according to expression patterns [76]. These transcripts were then partitioned into subclusters by cutting the hierarchically clustered tree at 50% height of the tree [76].

GO enrichment analysis was performed by running GOSeq on significantly differentially expressed transcripts [81]. Significantly enriched GO terms for each structure (FDR < 0.05) were then summarised using REVIGO with SimRel semantic similarities (0.5 allowed similarity) [82].

### 4.4. Analysis of Toxin-Like Transcripts

Transcripts with homology to known toxins were identified from BLASTp searches against the UniProt database by filtering for transcripts annotated with the ‘toxin activity’ GO term (GO:0090729), excluding bacterial toxins. To limit results to lineage-specific toxin-like transcripts, the translated ORFs generated by TransDecoder were then used as BLASTp queries against database of cnidarian toxins from ToxProt (accessed on 21 April 2021) using BLAST+ 2.3.0. Output from edgeR was then filtered to include only toxin-like transcripts and using Trinity PtR, heatmaps were generated and used to visualise differentially expressed toxin-like transcripts. Protein family designations for toxin-like transcripts were assigned using data available in the UniProt database and toxin families were further categorised by biological function as in previous work [83,84].

## Figures and Tables

**Table 1:** Transcriptome sequencing and assembly parameters for actiniarian species.

	*C. adhaesivum*	*D*. cf. *armata*	*H. hemprichii*	*M*. *doreensis*	*P*. *semoni*
Raw Reads	200.5 million	160.2 million	240.3 million	142.2 million	305.0 million
Transcripts	628,468	164,583	101,150	108,541	208,537
Genes	451,132	97,982	74,496	64,558	107,984
DE ^1^ Transcripts	3672	156	3118	271	1411
N50	609	1514	1370	1493	2823
E90 N50	1346	2068	1991	1549	3241
E90 transcripts	105,056	35,114	19,888	18,285	19,460
BUSCO ^2^	93.9%	97.2%	92.0%	88.9%	95.0%

^1^ DE; differentially expressed defined as greater than 4-fold expression change (false discovery rate [FDR] ≤ 0.05). ^2^ BUSCO; Benchmarking Universal Single-Copy Orthologs.

**Table 2:** Venom arsenal of five sea anemone species, *C. adhaesivum*, *D.* cf. *armata*, *H. hemprichii*, *M. doreensis* and *P. semoni,* according to biological function and toxin family. The values listed correspond to the number of toxin-like transcripts identified for each toxin family.

Category	ToxProt Family	*C. adhaesivum*	*D.* cf. *armata*	*H. hemprichii*	*M. doreensis*	*P. semoni*
Auxiliary	Peptidase M12A	8	3	1	4	35
Membrane-active	Actinoporin family	15	0	9	9	4
	Jellyfish toxin family	0	1	0	0	0
	MACPF toxin family	7	3	3	11	29
Mixed function enzymes	Phospholipase A2 family	21	12	10	11	15
Neurotoxin	Sea anemone type 3 (BDS) potassium channel toxin family	24	11	13	7	2
	Sea anemone type 1 potassium channel toxin family	6	2	3	6	2
	Sea anemone type 5 potassium channel toxin family	1	0	0	0	1
	Sea anemone sodium channel inhibitory toxin family	2	1	3	1	5
	Cnidaria small cysteine-rich protein (SCRiP) family	1	1	0	0	2
	Sea anemone short toxin (type III) family	1	0	1	0	0
	Sea anemone structural class 9a family	0	2	0	3	0
Protease Inhibitor	Venom Kunitz-type family	14	12	9	6	11
Unknown	EGF domain peptide family	2	0	1	2	1
	Sea anemone 8 toxin family	14	5	6	8	9
	Acrorhagin	2	3	1	1	0
	Unknown	0	5	0	3	0
		118	61	60	72	116

## Data Availability

Raw reads for *C. adhaesivum*, *H. hemprichii*, *M. doreensis* and *P. semoni* are available at the NCBI SRA under BioProject PRJNA715506. Accession numbers for *C. adhaesivum*, *H. hemprichii*, *M. doreensis* and *P. semoni* can be viewed in Supplementary Materials. Raw reads for *D.* cf. *armata* are registered in EMBL-ENA under project accession number PRJEB42903 and ENA sample runs as follows: DA4ITa—ERXS6629214, DA4OTc—ERS6629215, DA5IT—ERS6629216 and DA5OT—ERS6629217.

## Supplementary Materials

The following are available online at https://www.mdpi.com/article/10.3390/toxins13070452/s1, Figure S1: Heatmap of differentially expressed genes (centered FPKM values) for *C. adhaesivum* and *P. semoni*, Figure S2: Patterns of differentially expressed toxins for *D*. cf. *armata* and *M. doreensis*, Table S1: Sequence read archive and BioSample accession numbers.
