# Supplementary material for: Tentacle Morphological Variation Coincides with Differential Expression of Toxins in Sea Anemones
Source: Toxins (Basel). 2021 Jun 29;13(7):452. doi: 10.3390/toxins13070452 (PMC8310139; doi:10.3390/toxins13070452)
Supplement: Supplementary file 1 [file toxins-13-00452-s001.zip › toxins-1255243-supplementary.pdf]

Supplementary Materials: Tentacle Morphological Variation Coincides with Differential Expression of Toxins in Sea Anemones

Lauren M. Ashwood, Michela L. Mitchell, Bruno Madio, David A. Hurwood, Glenn F. King, Eivind A.B. Undheim, Raymond S. Norton and Peter J. Prentis

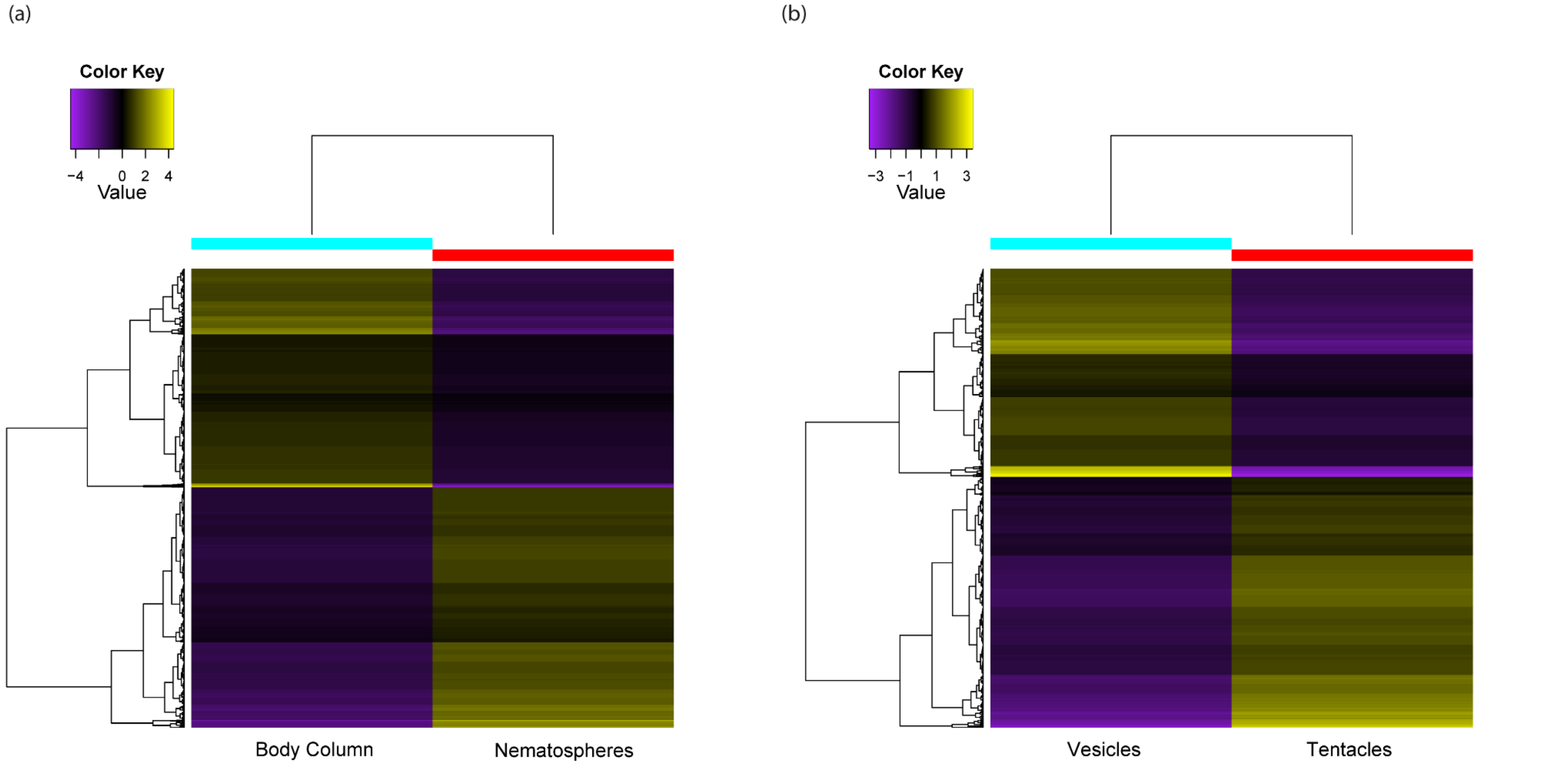

Figure S1. Heatmap of differentially expressed genes (centered FPKM values) for *C. adhaesivum* and *P. semoni*: (**a**) Heatmap of differentially expressed genes (centered FPKM values) for body volumn and nematospheres in *C. adhaesivum*; (**b**) Heatmap of differentially expressed genes (centered FPKM values) for vesicles and tentacles in *P. semoni*.

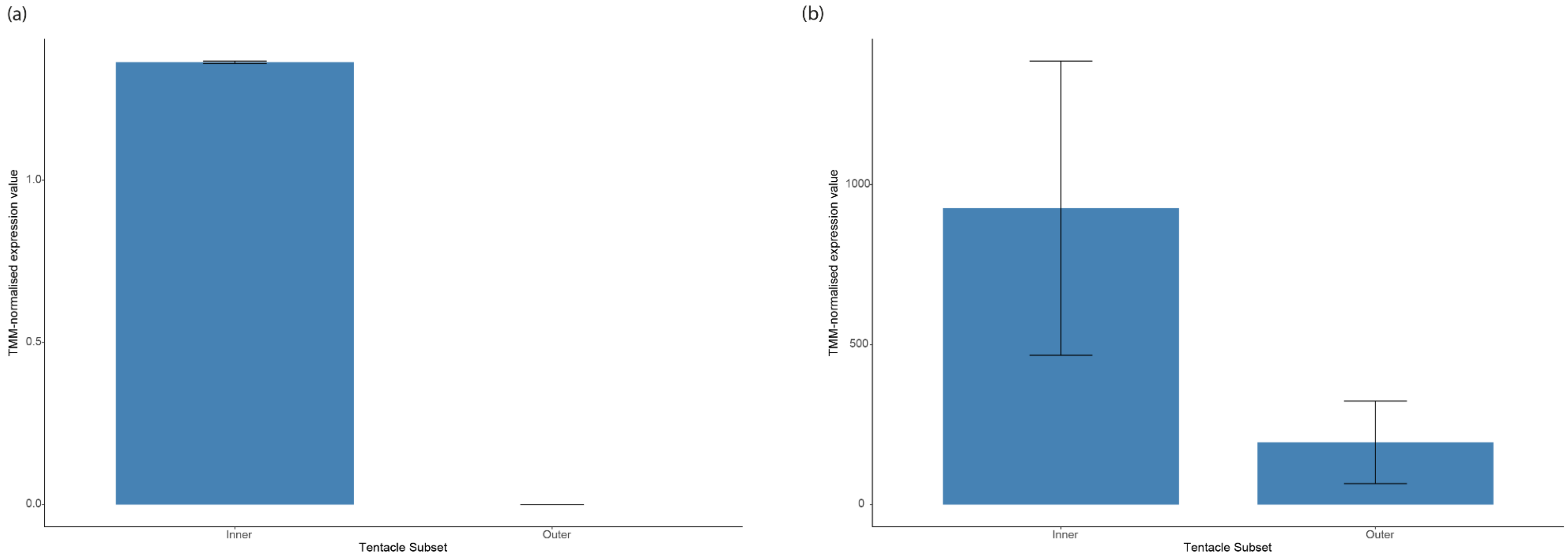

Figure S2. Patterns of differentially expressed toxins for *D.* cf. *armata* and *M. doreensis*. (**a**) Mean Trimmed Mean of M-values (TMM) expression values for transcript with homology to nematocyst expressed protein 6 for inner and outer tentacles in *D.* cf. *armata*, with error bars showing standard deviation; (**b**) Mean TMM-expression values for transcript with homology to δ-alicitoxin-Amc1a for inner and outer tentacles in *M. doreensis*, with error bars showing standard deviation.

Table S1. Sequence read archive and BioSample accession numbers. All samples for *C. adhaesivum*, *H. hemprichii*, *M. doreensis* and *P. semoni* were submitted under one BioProject (accession number PRJNA715506).

Species	Tissue	BioSample	SRA accession
C. adhaevisum	Body column	SAMN18353001	SRR14115233
C. adhaevisum	Nematospheres	SAMN18353002	SRR14115232
H. hemprichii	Body column	SAMN18353003	SRR14115229
H. hemprichii	Endocoelic tentacles	SAMN18353004	SRR14115228
H. hemprichii	Exocoelic tentacles	SAMN18353005	SRR14115227
H. hemprichii	Nematospheres	SAMN18353006	SRR14115226
M. doreensis	Inner tentacle, replicate 1	SAMN18353007	SRR14115225
M. doreensis	Inner tentacle, replicate 2	SAMN18353008	SRR14115224
M. doreensis	Outer tentacle, replicate 1	SAMN18353009	SRR14115223
M. doreensis	Outer tentacle, replicate 2	SAMN18353010	SRR14115222
P. semoni	Vesicles	SAMN18353011	SRR14115231
P. semoni	Tentacles	SAMN18353012	SRR14115230
